# Insight into the Global Negative Regulation of Iron Scavenger 7-HT Biosynthesis by the SigW/RsiW System in *Pseudomonas donghuensis* HYS

**DOI:** 10.3390/ijms24021184

**Published:** 2023-01-07

**Authors:** Shiyu Teng, Tingting Wu, Donghao Gao, Siyi Wu, Yaqian Xiao, Yan Long, Zhixiong Xie

**Affiliations:** Hubei Key Laboratory of Cell Homeostasis, College of Life Sciences, Wuhan University, Wuhan 430072, China

**Keywords:** *Pseudomonas donghuensis* HYS, SigW, anti-σ factor, negative regulator, siderophore, 7-hydroxytropolone

## Abstract

7-Hydroxytropolone (7-HT) is a unique iron scavenger synthesized by *Pseudomonas donghuensis* HYS that has various biological activities in addition to functioning as a siderophore. *P. donghuensis* HYS is more pathogenic than *P. aeruginosa* toward *Caenorhabditis elegans*, an observation that is closely linked to the biosynthesis of 7-HT. The nonfluorescent siderophore (*nfs*) gene cluster is responsible for the orderly biosynthesis of 7-HT and represents a competitive advantage that contributes to the increased survival of *P. donghuensis* HYS; however, the regulatory mechanisms of 7-HT biosynthesis remain unclear. This study is the first to propose that the ECF σ factor has a regulatory effect on 7-HT biosynthesis. In total, 20 ECF σ factors were identified through genome-wide scanning, and their responses to extracellular ferrous ions were characterized. We found that SigW was both significantly upregulated under high-iron conditions and repressed by an adjacent anti-σ factor. RNA-Seq results suggest that the SigW/RsiW system is involved in iron metabolism and 7-HT biosynthesis. Combined with the siderophore phenotype, we also found that SigW could inhibit siderophore synthesis, and this inhibition can be relieved by RsiW. EMSA assays proved that SigW, when highly expressed, can directly bind to the promoter region of five operons of the *nfs* cluster to inhibit the transcription of the corresponding genes and consequently suppress 7-HT biosynthesis. In addition, SigW not only directly negatively regulates structural genes related to 7-HT synthesis but also inhibits the transcription of regulatory proteins, including of the Gac/Rsm cascade system. Taken together, our results highlight that the biosynthesis of 7-HT is negatively regulated by SigW and that the SigW/RsiW system is involved in mechanisms for the regulation of iron homeostasis in *P. donghuensis* HYS. As a result of this work, we identified a novel mechanism for the global negative regulation of 7-HT biosynthesis, complementing our understanding of the function of ECF σ factors in *Pseudomonas*.

## 1. Introduction

*Pseudomonas* is a genus of Gram-negative bacteria widely found in the environment. Many members of this genus are significant pathogens in animals and plants. Obtaining nutrients from different species is necessary for the colonization and pathogenicity of pathogens, and iron is an essential mineral nutrient for almost all organisms. The pathogenicity of *Pseudomonas* is usually closely related to iron metabolism [1]. *Pseudomonas aeruginosa* is an important opportunistic pathogen that has aroused extensive research interest due to its increasing drug resistance, which is closely related to its strong biofilm formation [2,3]. Recent studies on *P. aeruginosa* PAO1 found that iron acquisition plays a key role in its biofilm formation and bacterial virulence [4]. Production of pyoverdine provides *P. aeruginosa* with a competitive advantage when co-cultured with *Staphylococcus aureus* and *Burkholderia cenocepacia* [5]. *P. syringae* MB03 can secrete pyoverdine and, thus, has the ability to capture more iron resources in an interaction model with *C. elegans* under iron-starvation conditions, which indirectly led to anoxia and the eventual death of *C. elegans.* Multiple studies have indicated that iron is a key virulence determinant in *P. syringae* MB03 [6]. Iron uptake and metabolism are involved in many important life processes and are tightly regulated, providing *Pseudomonas* species with a competitive advantage in the environment.

Siderophores are iron scavengers secreted by microorganisms and an important part of a key strategy used by bacteria to maintain intracellular iron homeostasis in iron-limited environments [7]. Pathogens also secrete siderophores in order to compete with their hosts for iron; however, the siderophores themselves are toxic to cells and so their biosynthesis is tightly regulated. One of the reasons why *Pseudomonas* can survive in a variety of complex environments is their abundance of siderophore species [8,9,10]. Pyoverdine is shown to be an important virulence factor in strains, including *P. aeruginosa* PAO1, *P. fluorescens*, and *P. syringae* pv. *tabaci* 6605 [11,12,13]. Pyoverdine is highly negatively correlated with iron concentration within a certain range, and its levels are strictly regulated by global factors. When the receptor FpvA on the outer membrane senses the ferripyoverdine complex, the signal is transmitted to the transmembrane anti-σ factor FpvR, thereby relieving the inhibition of the structural gene *pvdA* and further initiating the transcription of related genes [14]. The Gac/Rsm regulatory cascade establishes a connection with iron homeostasis by regulating pyoverdine biosynthesis in a variety of *Pseudomonas* species [15,16,17]. Notably, the components in the Gac/Rsm pathway and the controlled genes are similar, but the results of their regulation may be reversed [18]. It is worth mentioning that this system positively controls pyoverdine production in different *P. aeruginosa* strains, whereas it negatively regulates pyoverdine synthesis in *P. donghuensis* HYS [15,19,20,21]. In addition, *P. donghuensis* can secrete the nonfluorescent siderophore 7-Hydroxytropolone (7-HT), which can kill fungi and nematodes [22,23,24]. Previous reports have indicated that the biosynthesis of 7-HT is positively regulated by the Gac/Rsm system and LysR family transcription factors [15,19,25].

ECF σ factors play crucial roles in maintaining bacterial iron homeostasis [26,27]. In many pathogens, iron acquisition related to virulence and the expression of genes responsible for siderophore synthesis are regulated by ECF σ factors [28]. The core ECF σ factor of *P. aeruginosa*, PvdS, not only regulates the synthesis of pyoverdine but also affects the expression of protease through the Ret/Rsm pathway; these regulatory mechanisms are facilitated by iron [29,30]. Generally, the activity of ECF σ factors is regulated by cognate membrane-bound anti-σ factors. In the absence of an outer membrane stimulatory signal, the anti-σ factor FpvR persistently inhibits the activity of FpvI and PvdS, which are required for the synthesis of the receptor FpvA and pyoverdine, respectively [31]. *Brucella melitensis* M28 has been shown to encode an ECF16 σ/anti-σ system that is involved in regulating the expression of T4SS and some virulence genes [32]. In *Rhodobacter sphaeroides*, the activity of σ^E^ is inhibited by ChrR, a member of the zinc family that contains the anti-σ factor domain [33]. AlgU is a widely conserved ECF σ factor that regulates the expression of more than 800 genes in *P. syringae* [34]. AlgU and MucA of *P. aeruginosa* constitute an ECF σ/anti-σ system, where the MucA protein is typically necessary for survival but is no longer necessary in strains lacking AlgU [35]. The genomes of *P. aeruginosa* PAO1 and *P. putida* KT2440 encode 19 ECF σ factors, with most related research having focused on oxidative stress and virulence regulation [36,37]. *Pseudomonas* species can survive in a variety of environments, thanks to their rich and diverse metabolic regulatory systems [38]. Their complex regulatory networks, including ECF σ factors, enable them to respond positively to changes in environmental conditions, such as iron starvation and oxidative stress [39,40].

In recent years, *P. donghuensis* species have been found in different countries and regions [41,42]. As mentioned above, *P. donghuensis* HYS has been shown to be more toxic than *P. aeruginosa* toward *C. elegans* [22,43]. Furthermore, it can produce a specific iron scavenger, 7-HT, with a variety of biological activities. Therefore, the study of pathogenic mechanisms and 7-HT biosynthesis has aroused widespread interest among researchers. Additionally, 7-HT itself is directly highly toxic. Therefore, we focused on transcription factors whose transcription levels are elevated under high ferrous ion concentrations, as this is often an indication that they are often involved in the negative regulation of 7-HT synthesis. In this paper, through genome-wide scanning, 20 putative ECF σ factors were identified in *P. donghuensis* HYS. An ECF σ/anti-σ system pair was found to actively participate in the iron response, and we attempted to explore the mechanism by which SigW/RsiW is involved in iron metabolism in *P. donghuensis* HYS. The results of this investigation can supplement current understanding of the complex mechanisms of iron metabolism in *Pseudomonas*.

## 2. Results

### 2.1. Response of ECF σ Factors to Extracellular Ferrous Iron in P. donghuensis HYS

**Figure 1:**
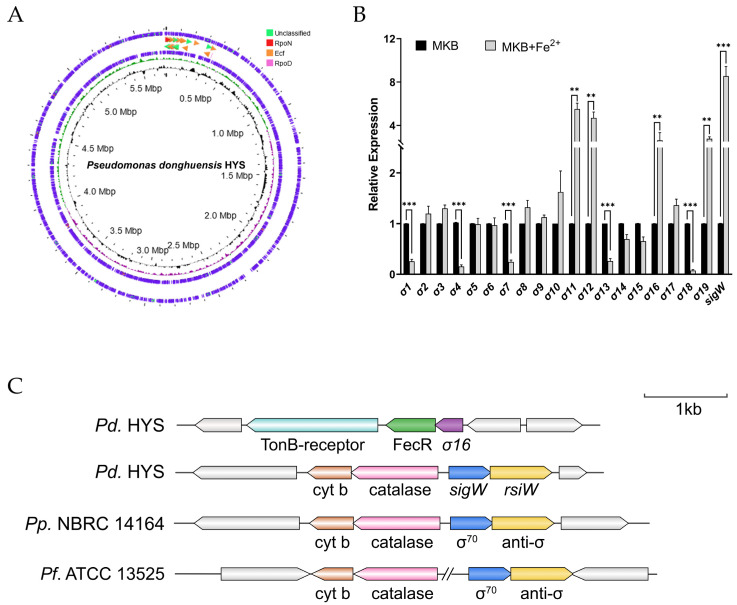
Screening of the ECF σ factors involved in iron metabolism in *P. donghuensis* HYS. RNA was isolated from the indicated strains grown to the exponential phase at 30 °C in liquid MKB culture with or without 30 μM FeSO_4_·7H_2_O supplementation. The error bars indicate the mean ± SD of three independent experiments. Statistical significance was calculated using one-way ANOVA Dunnett’s multiple comparison test, ** *p* < 0.001; *** *p* < 0.0001. (**A**) The *P. donghuensis* strain HYS σ factors regulome. (**B**) Expression of 20 ECF σ factors encoded on the genome in different extracellular iron environments. (**C**) Genetic organization of *σ16* and *sigW*. The locus tags of the corresponding genes in *P. putida* NBRC 14164 (accession number NC_021505) and *P. fluorescens* ATCC 13525 (accession number NZ_LT907842.1) are shown under the arrows.

To investigate whether σ factors are involved in iron metabolism and siderophore synthesis in *P. donghuensis* HYS, we scanned the entire genome sequence in the P2TF database. The identity value was set as greater than 80% and 35 σ factors were predicted, 20 of which corresponded to the sub-family of extracytoplasmic function (ECF) σ factors, 3 were RpoD family members, 1 was an RpoN family member, and the remaining 11 were unclassified σ factors, according to the gene annotations (Figure 1A, Table S2). To determine whether the ECF σ factors respond to the stimulation of extracellular ferrous ions, changes in the transcription levels of the 20 ECF σ factors under iron-limited and -rich conditions were detected using qRT-PCR. The results demonstrate that *σ1*, *σ4*, *σ7*, *σ13*, and *σ18* were significantly downregulated in response to the extracellular addition of 30 μM ferrous ions. In addition, five ECF σ factors were significantly upregulated, including *σ11*, *σ12*, *σ16*, *σ19*, and *sigW* (Figure 1B).

In order to explore the mechanisms of iron metabolism and siderophore synthesis, we then focused on identifying which ECF σ factors had elevated transcription levels. Combined with the results of genome annotation and mapping, the results indicated that *σ11* and *σ19* are housekeeping genes encoding RpoE and RpoH, respectively, *σ12* encodes a FecR domain protein involved in iron uptake, and the other two (*sigW* and *σ16*) encode members of the σ^70^ family, whose functions are unknown and thereby caught our attention (Figure S1, Table S2). The qRT-PCR data show that *sigW* is the most upregulated following stimulation with ferrous ions, with an 8.5-fold increase in expression compared with its levels in the control group.

Genomic localization showed that *σ16* is also adjacent to a gene encoding FecR protein, which may be synergistically involved in iron uptake and transmembrane transport, while *sigW* is adjacent to a downstream anti-σ factor named *rsiW*, which has also been found in *P. putida* NBRC 14164 and *P. fluorescens* ATCC 13525. It has been shown that this structure is conserved in the group of *P. fluorescens* DNA homologues (Figure 1C). Thus, by assessing the response to extracellular ferrous ions, we found that the ECF-σ/anti-σ pair operates as a system involved in iron metabolism in *P. donghuensis* HYS.

### 2.2. SigW Is Only Regulated by RsiW under Iron-Limiting Conditions in P. donghuensis HYS

**Figure 2:**
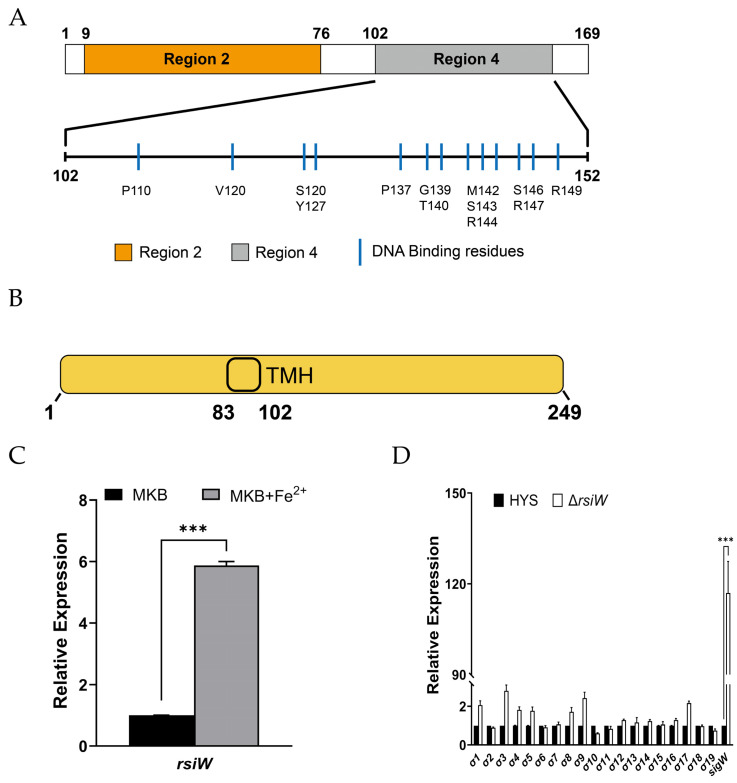
RsiW functions as the anti-σ factor of SigW in *P. donghuensis* HYS. The domain architectures are illustrated, along with their structures. The region 2 and region 4 domains usually carried by ECF σ factors are shown. The blue lines represent DNA binding sites with the corresponding amino acid indicated below (**A**). The transmembrane helices (TMH) in RsiW were predicted by the transmembrane protein topology prediction tool TMHMM (**B**). (**C**) Expression of the anti-σ factor, *rsiW* in different extracellular iron environments. MKB simulates an iron-limited environment and MKB supplement 30 μM ferrous ions simulates an iron-rich environment. (**D**) The relative expression of 20 ECF σ factors in the wild-type strain of *P. donghuensis* HYS and Δ*rsiW* mutant under the iron-limited conditions. RNA was isolated from the indicated strains grown to the exponential phase at 30 °C in liquid MKB culture with or without 30 μM FeSO_4_·7H_2_O supplementation. The error bars indicate the mean ± SD of three independent experiments. Statistical significance was calculated using one-way ANOVA Dunnett’s multiple comparison test, *** *p* < 0.0001.

The arrangement of *sigW* and *rsiW* on the genome of *P. donghuensis* HYS conforms to the functional model mediated by the ECF σ/anti-σ system in *Pseudomonas* (Figure 1C). Bioinformatic analysis of SigW, consisting of 169 amino acids, and its downstream anti-σ factor named RsiW, comprising 249 aa, was conducted. Further prediction revealed that SigW contains two conserved domains—regions 2 and 4—which are the most-conserved domains of the ECF σ factors in the σ^70^ family and can selectively regulate the transcription of specific genes. The InterPro Online website was used to predict the DNA binding sites for SigW, all of which clustered in region 4 (Figure 2A). The anti-σ factor in bacteria is usually a one-way transmembrane protein, which is responsible for transferring extracellular signal stimulation to the intracellular σ factor, thus, regulating the transcription of related genes. RsiW is an anti-σ factor that contains a transmembrane region between positions 83 and 102 (Figure 2B). Moreover, the relative expression of *rsiW* was measured in an MKB medium, supplemented or not with 30 µM ferrous ions, showing that the expression of *rsiW* was also upregulated after stimulation with extracellular ferrous ions (Figure 2C). In Δ*rsiW* mutant strains, the expression of *sigW* is significantly upregulated by more than 100-fold under the same culture as above. We also found that *sigW* is the only ECF σ factor in the whole genome inhibited by *rsiW* in an iron-limited environment (Figure 2D). These results demonstrate that the SigW/RsiW pair functions as an ECF σ factor/anti-σ factor system involved in iron metabolism in *P. donghuensis* HYS.

### 2.3. The SigW/RsiW System Is Involved in Iron Metabolism and 7-HT Biosynthesis

**Figure 3:**
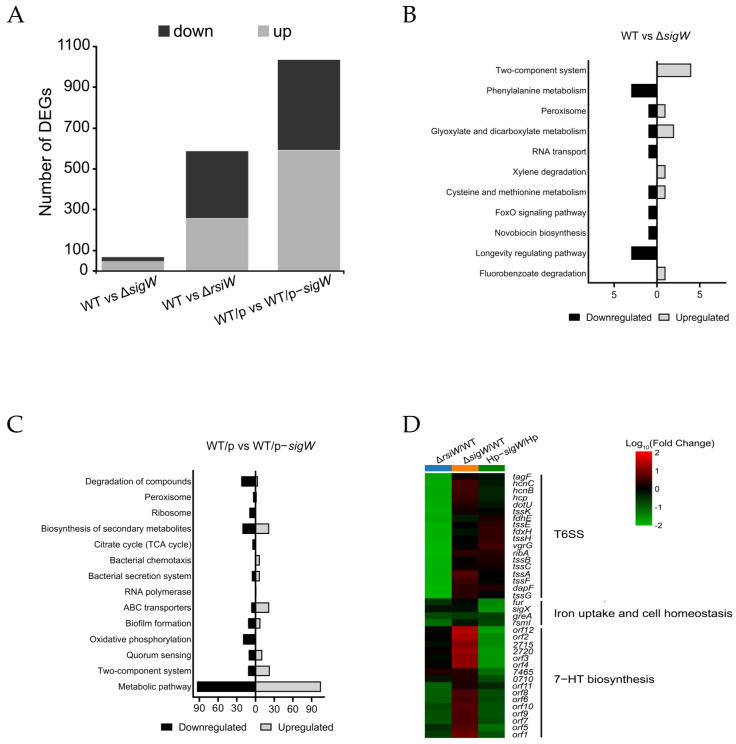
Transcriptional profiling with RNA sequencing identified SigW/RsiW regulated genes in *P. donghuensis* HYS. Wild-type strains lacking *sigW* or *rsiW* or carrying pBBR2-*sigW* (overexpressing *sigW*), grown in MKB medium were analyzed by RNA sequencing. (**A**) Stacking diagram representing the number of differential genes among the three groups. The black and grey bars represent down- and upregulated genes, respectively. In the three comparison groups, the total number of differential genes was 66, 585, and 1032, respectively. The graph was created based on the mean value of fold changes in triplicates. Kyoto Encyclopedia of Genes and Genomes (KEGG) analysis of differentially expressed genes (**B**) (Δ*sigW* vs. HYS) and (**C**) (HYS/pBBR2−*sigW* vs. HYS/pBBR2). The black and grey bars represent down- and upregulated genes, respectively, while the bars represent the number of genes related to that pathway. Selection of enrichment pathways with *p* values less than 0.05 is shown in the histogram. *p*-values < 0.05 indicate that the function was significantly enriched. (**D**) Heatmap demonstrating a selection of genes differentially expressed in the comparisons are indicated above each column. The diagram was based on the mean value of fold changes in triplicates.

To obtain a comprehensive understanding of the cellular functions of the SigW/RsiW system in P. donghuensis HYS, target genes of SigW/RsiW were determined by transcriptome sequencing (RNA-Seq). We extracted total RNA grown to mid-exponential phase in MKB medium with three biological replicates per sample. Three comparison groups were set up, namely ΔsigW versus WT, ΔrsiW versus WT, and HYS/pBBR1-MCS2-sigW versus HYS/pBBR1-MCS2. WT denotes that wild-type strain of P. donghuensis HYS, ΔsigW, and ΔrsiW denotes the single mutants with deletion of in-frame, respectively, and they are both single copy genes. HYS/pBBR1-MCS2 means wild-type strain containing an empty vector, which is abbreviated as HYS/pBBR2 in the following. Meanwhile, HYS/pBBR2-sigW refers to sigW being overexpressed in the wild type and the expression ploidy is increased by about 400-fold according to the qRT-PCR assay (Figure S2). Using RNA-Seq, we compared the mRNA levels in the mid-exponential culture prepared from the ΔsigW and ΔrsiW mutant strains expressing WT or overexpressing sigW from a plasmid. A 5-fold change was chosen as the cutoff point (FC > 5). Analysis of the whole transcriptional profile data showed that 66, 585, and 1032 genes were differentially expressed in the binary comparison of ΔsigW versus WT group, ΔrsiW versus WT group, and HYS/pBBR1-MCS2-sigW versus HYS/pBBR1-MCS2 group, respectively. Stacking diagrams show that the number of differentially down- and upregulated genes in each group are 18 and 48, 330 and 255, and 442 and 590, respectively (Figure 3A).

The absence of *sigW* modulated 66 RNA species, with 48 up- and 18 downregulated, while the overexpression of *sigW* regulated 1032 RNA species, with 590 up- and 442 downregulated, which code for proteins involved in a variety of processes in *P. donghuensis* HYS, including metabolic pathways, two-component systems, bacterial secretion systems, biofilm formation, biosynthesis of secondary products, and quorum sensing (Figure 3B,C). The functions of almost half of the affected transcripts, however, are unknown.

In order to further clarify which biological processes are controlled in vivo by the SigW/RsiW system, the clustering of multiple differentially expressed genes was conducted. The heatmap shows the results for functional clustering of differentially expressed genes, excluding some hypothetical proteins. Following screening for genes with a difference in expression greater than or equal to a 5-fold change (FC ≥ 5), the resulting gene clusters can be roughly divided into three groups. The first group is of members of the bacterial type VI secretion system (T6SS) involved in the synthesis and transport of proteins. Genes encoding components of T6SS were uniformly downregulated by at least 5-fold in Δ*rsiW* mutants compared with wild-type. The second group includes four regulatory factors associated with iron uptake and cell homeostasis, all of which were significantly downregulated in *sigW*-overexpressed samples. Strikingly, the third group consisted of 12 ORFs on the The nonfluorescent siderophore (*nfs*) gene cluster, which is involved in 7-HT biosynthesis in *P. donghuensis* HYS, and these ORFs were upregulated in the absence of *sigW* and downregulated under *sigW* overexpression (Figure 3D). Taken together, the RNA-Seq data clarify that the SigW/RsiW system is, indeed, involved in the regulation of iron metabolism and the biosynthesis of 7-HT in *P. donghuensis* HYS.

### 2.4. SigW Inhibits the Production of Two Siderophores: Pyoverdine and 7-HT

**Figure 4:**
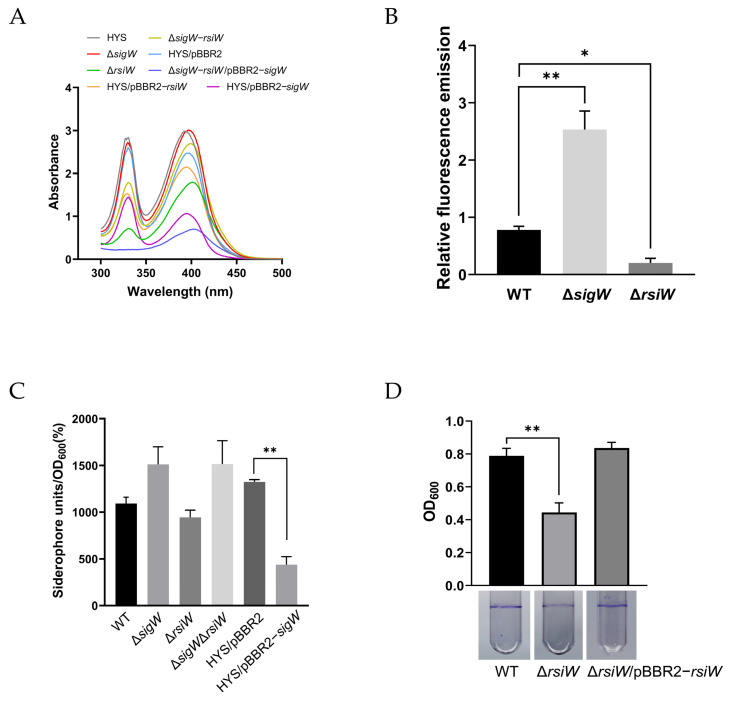
Effects of *sigW* and *rsiW* on siderophores and biofilm formation in *P. donghuensis* HYS. (**A**) Absorption spectra of the filtered supernatants of 24 h MKB cultures from wild-type HYS and the derivative strains. 7-HT has characteristic absorption at 330 and 392 nm, and pyoverdine has characteristic absorption at 405 nm. (**B**) Pyoverdine production in 24 h MKB cultures of wild-type HYS, Δ*sigW*, and Δ*rsiW* mutants. (**C**) Siderophore production in wild-type HYS and the derivative strains in liquid MKB medium was determined as siderophore units (percent) by the CAS liquid assay. (**D**) Deletion of *rsiW* decreased biofilm formation. The biofilm formation of wild-type HYS, Δ*rsiW* mutant, and the complemented strain was detected using crystal violet staining (lower) and quantified b optical density measurement (upper). The error bars indicate the mean ± SD of three independent experiments. Statistical significance was calculated using one-way ANOVA Dunnett’s multiple comparison test, * *p* < 0.01; ** *p* < 0.001.

Siderophores are low-molecular-weight compounds produced by microorganisms in iron-deficient environments. HYS yields a nonfluorescent siderophore, 7-HT, in iron-restricted environments, which has characteristic absorbance at 330 and 392 nm, and another fluorescent siderophore, pyoverdine, which has characteristic absorbance at 405 nm. After 24 h of culture in MKB medium (simulating an iron-limited environment), the supernatant was collected for siderophore content detection [44]. The absorbances of 7-HT were remarkably lower in the Δ*rsiW* and Δ*sigW*–*rsiW* (pBBR2-*sigW*) mutants as well as in HYS/pBBR1-MCS2–*sigW* (Figure 4A), indicating that high expression of *sigW* inhibits the synthesis of 7-HT. Meanwhile, it was also slightly decreased in the Δ*sigW*–*rsiW* strains, and we suggest that this decrease may be due to the fact that *rsiW* deletion disrupts the transport function on the membrane and, therefore, detectable extracellular 7-HT secretion is reduced. However, we found that the production of pyoverdine was increased in the Δ*sigW* mutant, whereas the decrease in pyoverdine level was more pronounced in the Δ*rsiW* mutant (Figure 4B). Moreover, the absence of *sigW* led to significantly increased production of siderophores (Figure 4C). The RNA-Seq results in the previous section indicated that *rsiW* affects the expression of genes related to T6SS, which are related to biofilm formation, as confirmed by assays using crystalline violet showing a reduction in biofilm formation in the Δ*rsiW* mutant strain (Figure 4D). The results presented here indicate that *sigW* negatively affects 7-HT biosynthesis, while, in addition to inhibiting SigW, RsiW can also positively affect biofilm formation in *P. donghuensis* HYS.

### 2.5. SigW Directly Regulates the Expression of the nfs Cluster

**Figure 5:**
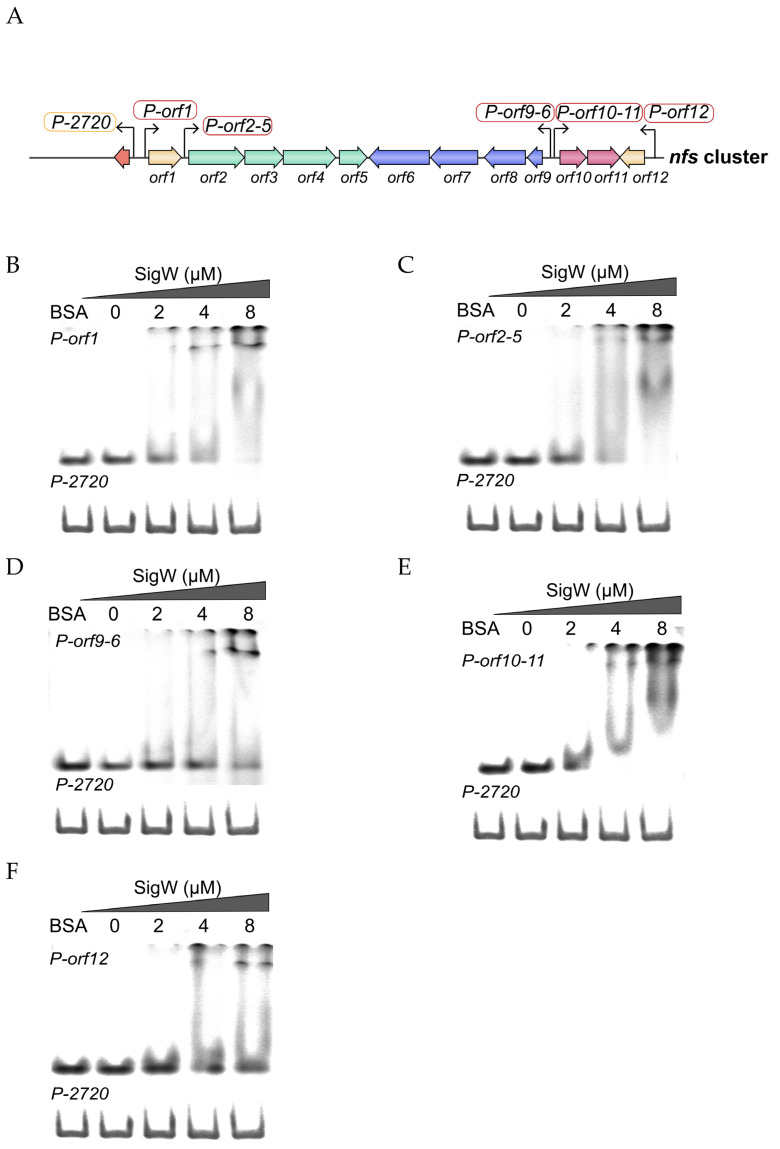
SigW binds directly to promoters on the *nfs* cluster for regulatory action. Localization map of promoters on the *nfs* cluster (**A**). The electrophoretic mobility shift assay shows that SigW binds to the promoter region of the wild-type (**B**) *orf1*, (**C**) *orf2*–*5*, (**D**) *orf9*–*6*, (**E**) *orf10*–*11*, and (**F**) *orf12,* respectively. Each reaction mixture contained 0.3 μM PCR products of the wild-type *orf1*–217 to –1, *orf2*–191 to –1, *orf9*–225 to –1, *orf10*–237 to –1, *orf12*–246 to –1, and *PD2720*–210 to –1. The protein concentrations are indicated above the lane. BSA and *P-2720* were used as negative controls. Data are representative of three independent replicates.

ECF σ factors can bind to the promoter of target genes and play a regulatory role. Key genes associated with the 7-HT biosynthesis in *P. donguensis* HYS form one cluster, named the *nfs* cluster, consisting of five operons. SigW contains a conserved DNA-binding domain at its C-terminus (Figure 2A). To examine whether SigW regulates the expression of *nfs* cluster genes, the purified SigW and promoter regions of *orf1*, *orf12*, *orf2–orf5*, *orf9–orf6*, and *orf10–orf11* were used to ascertain protein–DNA interactions using EMSAs. A pattern diagram shows the positioning of promoters on the *nfs* cluster, with the same colors representing that they are co-transcribed units (Figure 5A). The promoter sequences of the *nfs* cluster were predicted using the PromPredict online tools.

ORF1 and ORF12 are LysR and TetR/AcrR-type regulators that regulate 7-HT biosynthesis [25]. SigW efficiently bound the promoter regions of *orf1* and *orf12*, resulting in gel mobility shift (Figure 5B,F).

*orf2*–*orf5* is the second operon in the *nfs* cluster. The EMSA results indicate that SigW weakly binds to the promoter region of this operon (Figure 5C). The normal transcription and expression of the *orf9–orf6* operon directly determines whether 7-HT is synthesized. EMSA was used to verify that SigW binds to the promoter region of this operon (Figure 5D). *orf10* and *orf11* are co-transcription units associated with the synthesis of 7-HT. The results show that SigW can also bind to their promoters (Figure 5E). Under the same experimental conditions, we used BSA as a standard protein and the promoter region of the gene upstream of *orf1* numbered UW3_RS0102720 as two negative controls to demonstrate that the specificity of SigW in binding to the five operons. In order to determine whether the SigW regulates *nfs* cluster, we detected *nfs’– ‘lacZ* translational fusion expression in the *P. donghuensis* HYS or Δ*sigW* mutant under MKB culture conditions. The activity of promoters was increased in Δ*sigW* mutant compared to wild-type HYS (Figure S3). Overall, these results confirm that the expression of genes in the *nfs* cluster is directly regulated by SigW. SigW was able to specifically bind to the promoter regions to produce further regulatory effects.

**Figure 6:**
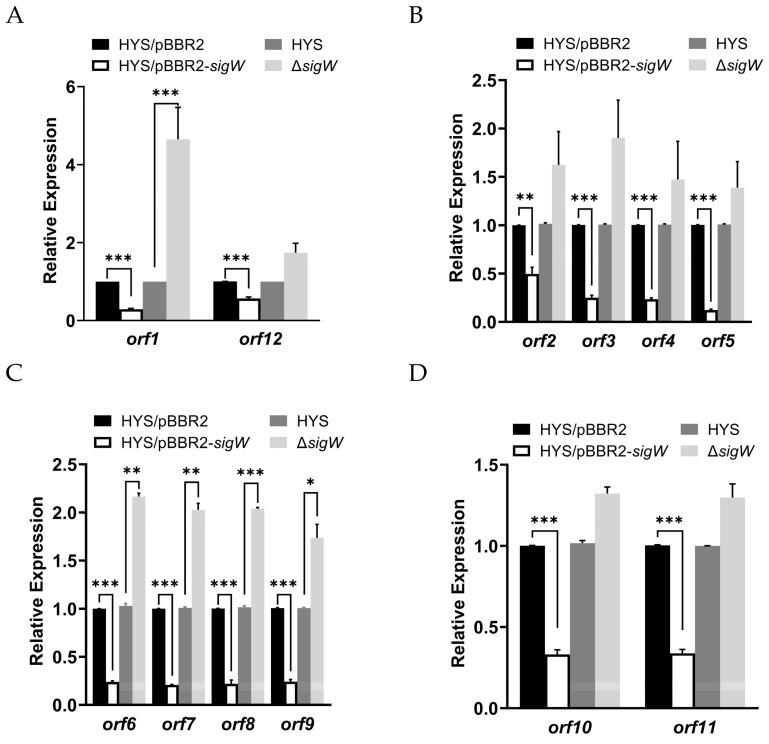
Validation of the nfs cluster regulated by SigW at the transcriptional levels. The results show the relative expression levels of the *orf1* and *orf12* (**A**), *orf2*–*orf5* (**B**), *orf9*–*orf6* (**C**), *orf10*–*orf11* (**D**) in HYS/pBBR2, HYS/pBBR2-*sigW*, Δ*sigW* mutant, and wild-type HYS. The transcriptional levels are shown as the relative expression of genes compared to the expression of the *rpoB* gene in various samples at the exponential phase, as measured by qRT-PCR. Error bars indicate the mean ± SD of three independent experiments. Statistical significance was calculated using one-way ANOVA Dunnett’s multiple comparison test, * *p* < 0.01; ** *p* < 0.001; *** *p* < 0.0001.

In order to further determine the regulatory mode of SigW on *nfs* cluster, the transcriptional levels of the five operons in the deletion and overexpression strains were detected by the qRT-PCR method. Consistent with the RNA-seq data, the expression levels of the five operons were significantly downregulated without exception when *sigW* was overexpressed, while their expression levels were increased in the Δ*sigW* strains (Figure 6). To sum up, these results indicated that SigW represses *nfs* cluster expression directly under the iron-restricted environment.

### 2.6. Transcription Start Sites for the SigW Operon

**Figure 7:**
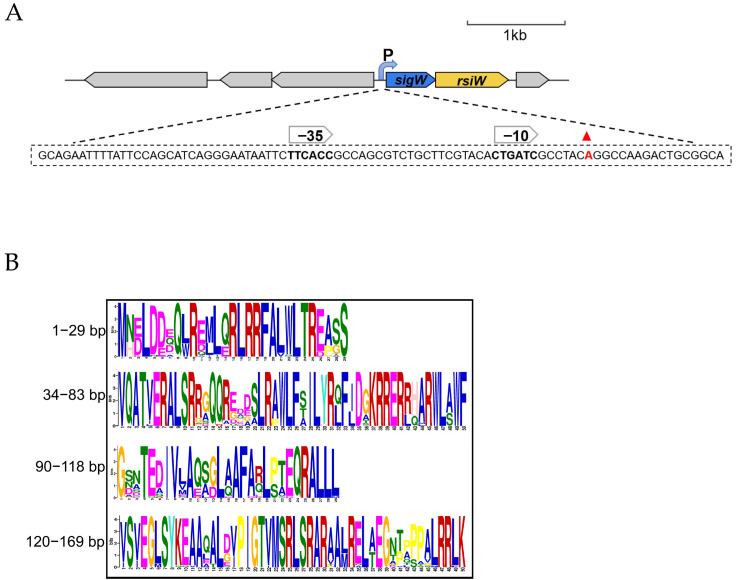
Genetic organization and characteristics of the *sigW* operon. (**A**) The 5′-RACE method was used to identify the TSS of the *sigW* operon using RNA sample, with the −10 and −35 motifs then deduced afterwards. The red triangle represents the start codon. The genes are drawn to scale. (**B**) MEME online prediction of the conserved motifs of SigW. The seqlogo plot shows how well the motif is conserved at each position; the higher the letter, the better the position is conserved. Different amino acids in the same position are scaled according to their frequency. The rules for construction logos are given B–C or G or T, Y–C or T, S–G or C, D–A or G or T, W–A or T, K–G or T. This graph is based on a motif sequence with an E-value less than or equal to 0.05. Further, MEME-Suite was used to predict motif information in the SigW sequence, as conserved motifs on transcription factors are usually involved in important biological processes. By submitting 20 amino acid sequences with >80% identity to SigW online, the output resulted in four highly conserved motifs with the second motif (34–83 bp) and fourth motif (120–169 bp) located in conserved regions 2 and 4, respectively, of the σ^70^ family in *Pseudomonas* (Figure 7B). In summary, the transcription structure information of this operon was clarified, and prediction of the −10, −35 regions, and conserved motifs is helpful for subsequent functional verification. In particular, the predicted conserved motifs clarified that this manipulator has a structure that is typical of the ECF σ factors.

We predicted one promoter and the corresponding −10 box (TCGTACACT) and −35 box (TTCACC) within 300 bp of the *sigW* start codon using the online tool BPROM on the Softberry website. In order to further clarify the structure of this operon, the transcription start site (TSS) for *sigW* was determined using the 5′-RACE method. Two gene-specific primers, GSP1 and GSP2, were used to amplify their 5′ ends using cDNA as a template. After amplification, bands of similar length to the target product were selected for sequencing, and the correct transcription start site was determined by comparison of multiple sequencing results. After aligning the amplified sequence with the 5’UTR sequence of the target gene, it was concluded that the A is located 17 bp upstream of the start codon is the TSS. The software predictions show general agreement with the experimental results, and the structure of the operon was determined, from which −10 (CTGATC) and −35 (TTCACC) regions were accordingly deduced (Figure 7A).

### 2.7. SigW Negatively Regulates the Gac/Rsm System to Inhibit 7-HT and Enhance Pyoverdine Production

**Figure 8:**
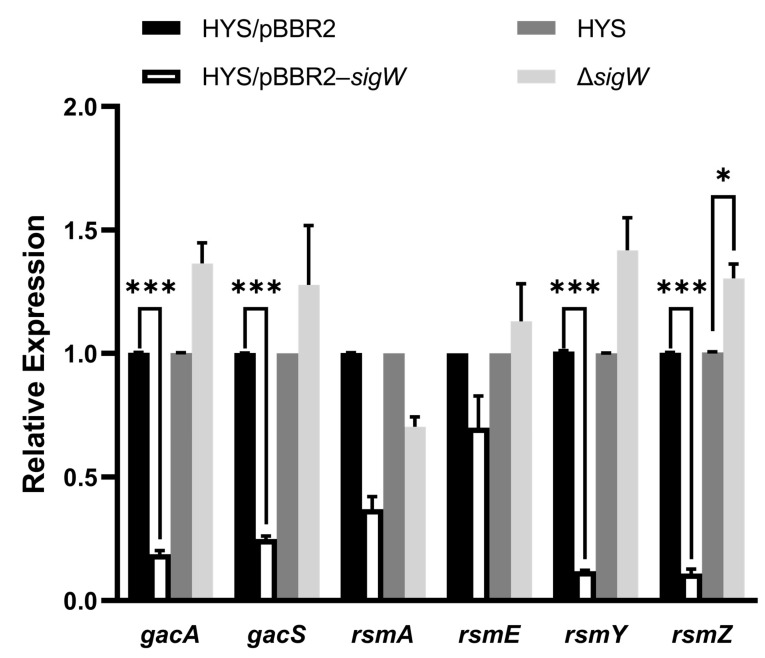
The regulatory relationship between SigW and the Gac/Rsm cascade system. Expression of *gacA/S*, *rsmA/E*, and *rsmY/Z* in *P. donghuensis* HYS. RNA was isolated from the indicated strains grown to the exponential phase at 30 °C in liquid MKB culture. Error bars indicate the mean ± SD of three independent experiments. Statistical significance was calculated using one-way ANOVA Dunnett’s multiple comparison test, * *p* < 0.01; *** *p* < 0.0001.

The Gac/Rsm cascade system varies widely among bacterial species, usually involving the ability to store and manage carbon as well as the expression of virulence or biological control factors. The Gac/Rsm cascade positively regulates 7-HT biosynthesis and negatively affects pyoverdine production in *P. donghuensis* HYS [19]. To explore the regulatory relationship between SigW and the Gac/Rsm system, qRT-PCR was used to verify the mRNA expression levels of *gacS*, *gacA*, *rsmY*, *rsmZ*, *rsmA,* and *rsmE*, which are key genes in the Gac/Rsm system, in *sigW* deleted and overexpressed strains. The results show that overexpression of *sigW* significantly reduces the expression of *gacA*, *gacS*, *rsmY*, and *rsmZ*, whereas deletion of *sigW* enhances the expression of these genes (Figure 8). These results indicate that *sigW*, as a global regulatory factor, has a negative regulatory effect on the Gac/Rsm system in *P. donghuensis* HYS. In particular, SigW inhibits the Gac/Rsm system, which may be one of the routes by which it inhibits 7-HT synthesis. However, this needs to be verified by more in-depth research. The negative effect of SigW on the Gac/Rsm system and the elevated pyoverdine production detected in the Δ*sigW* strain suggest that the Gac/Rsm system is indeed not directly regulated in relation to pyoverdine synthesis in *P. donghuensis* HYS, which is consistent with the findings of Yu et al. [19].

## 3. Discussion

Bacteria chelate ferric ions mainly by secreting siderophores in iron-limited environments; meanwhile, in iron-rich environments, they inhibit siderophore synthesis to avoid the burden of excess iron intake on cells [8,45]. In other words, the biosynthesis of siderophores is tightly regulated in microorganisms. ECF σ factors mediate important iron-regulation mechanisms in *Pseudomonas*. As a vital ECF σ factor in *P. aeruginosa*, PvdS plays a crucial role in the regulation of pyoverdine biosynthesis. According to previous research, 7-HT—as a newly reported bacterial siderophore with antifungal and nematocidal functions—is a virulence factor of *P. donghuensis* [22,23,24,43,46]. Therefore, we aimed to focus more attention on some negative regulators in 7-HT synthesis, which, thus, play irreplaceable roles in iron homeostasis. To the best of our knowledge, the regulation of 7-HT biosynthesis by ECF σ factors has not been previously reported. It has been shown that bacterial σ factors, eukaryotic TFIIB, and archaeal TFB are all homologous and responsible for fine-tuning of transcription regulation in the domain of life [47]. There is also a significant correlation between the bacterial lifestyle and the number of ECF σ factors, where bacteria with complex lifestyles usually have more ECF σ factors [2,40]. *P. aeruginosa* PAO1 and *P. putida* KT2440 are widely distributed pathogenic microorganisms, and 19 ECF σ factors are found to be encoded within each of their genomes [36,37]. In contrast, the genome of *P. donghuensis* HYS encodes 20 ECF σ factors, 10 of which are significantly responsive to iron and most of which cooperate with FecR proteins, which is a relatively common transmembrane transport process involved in iron acquisition [48]. Two ECF σ factors of the σ^70^ family, named *sigW* and *σ16,* attracted our attention. In terms of their localization on the genome, *σ16* is adjacent to the gene encoding FecR protein. It is worth mentioning that SigW and RsiW are adjacent, and this arrangement has also been found in *P. putida* and *P. fluorescens*, which are the closest relatives to *P. donghuensis*, but no relevant features have yet been reported (Figure 1C). 7-HT is closely related to the pathogenicity of *P. donghuensis* HYS, so mechanisms for its regulation are strict and precise. We expected that RNA-Seq analysis would enable us to determine not only the iron-uptake mechanisms but also those genes whose expression is regulated directly by SigW/RsiW in the whole genome. Through clustering analysis of differentially expressed genes, we found that this system is closely related to the biosynthesis of 7-HT and T6SS (Figure 3D). These bioinformatic results promoted further exploration of the role of the SigW/RsiW system in the regulation of iron metabolism in *P. donghuensis* HYS.

Five operons on the *nfs* cluster play key roles in the synthesis, transport, and regulation of 7-HT. It has been found that the Gac/Rsm cascade system and LysR/TetR two-component system positively regulate the biosynthesis of 7-HT [19,25]. The concentration of iron and glycerol also affects 7-HT biosynthesis [24,46]. qRT-PCR showed that 12 ORFs in the *nfs* cluster could be inhibited by SigW and, in general, ECF σ factors may bind to the promoter of the target gene, playing a regulatory role. Therefore, an EMSA experiment was designed, and the results show that SigW binds to the promoters of the five operons. Taken together, these results suggest that SigW has a direct regulatory effect on the *nfs* cluster, and this pattern of inhibition of gene expression by binding to the promoter region may be due to changes in the structure of DNA, which makes RNA polymerase unable to function normally. We speculate that *sigW* overexpression increases its own activity and leads to the toxic expression of autoregulators. In addition, as the possibility of RNA polymerase interacting with RpoD is limited, overexpression of *sigW* will reduce the expression of the housekeeping gene *rpoD*, thereby reducing gene transcription and affecting the synthesis of intracellular metabolites, such as 7-HT. The common ECF σ/anti-σ system in *Pseudomonas* is the model cell surface signaling (CSS) system, which participates in the stress response, iron scavenging, and virulence [49]. Usually, the same anti-σ factor can control the activity of multiple σ factors, and the activation of σ factors can promote the expression of structural genes [50,51]. In particular, however, it was verified that the only ECF σ repressed by RsiW in the whole genome was SigW. SigW is able to repress the expression of not only structural genes responsible for 7-HT synthesis but also regulatory proteins, a pattern of global negative regulation not yet observed in *Pseudomonas*. Normally, *sigW* and *rsiW* may have self-regulatory effects. When *rsiW* is lost, *sigW* cannot be inhibited, resulting in its high expression and, thus, inhibiting the normal activities of cells.

**Figure 9:**
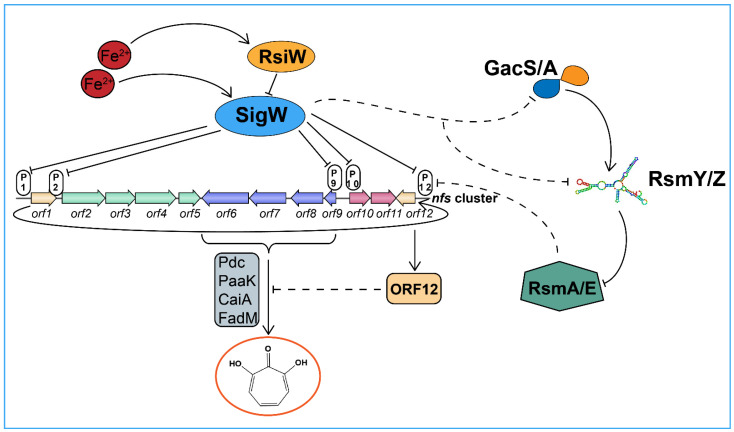
Schematic overview of the regulatory network of SigW/RsiW in *P. donghuensis* HYS. The T-shaped lines represent the negative control, the arrows represent the positive control, the solid lines highlight the existence of an already demonstrated regulation, and the dotted lines indicate the connections that were not confirmed in this work. P1, P2, P9, P10, and P12 represent the promoters of the five operons in the *nfs* cluster, respectively. ORF6, ORF7, ORF8, and ORF9 encode the Pdc, PaaK, CaiA, and FadM family proteins, respectively, which catalyze the key reaction of 7-HT biosynthesis.

We depict the biological function of the SigW/RsiW system in *P. donghuensis* HYS with the conceptual diagram shown in Figure 9. It has been confirmed that RsiW, as a one-way transmembrane protein, can respond in the regulation of ferrous ions and has a significant inhibitory effect on intracellular SigW in addition to T6SS and biofilm formation. SigW is a typical member of the σ^70^ family. Our results demonstrate that SigW can directly and negatively regulate the *nfs* cluster related to 7-HT, thus, affecting 7-HT biosynthesis, and has negative regulatory effects on the Gac/Rsm cascade system and LysR/TetR two-component system. However, there are still some problems that remain to be resolved. There are two possible reasons for the lack of increased 7-HT production in Δ*sigW* mutants (Figure 4A): On one hand, the toxicity of 7-HT itself could increase the burden on cells such that the host will actively operate a self-protection mechanism. On the other hand, it may not be able to create a strictly limited iron environment to continuously promote 7-HT production. In general, the Gac/Rsm system acts as a global switch in *Pseudomonas* to control the expression of multiple virulence and biological control factors, and there are often many transcription factors located downstream of Gac/Rsm and regulated by it, such as LysR and TetR/AcrR in *P. donghuensis* HYS [21,25]. However, our study shows that SigW is not regulated by Gac/Rsm but is located upstream of it and exerts a repressive effect, and the period during which this regulation is established may be related to the iron environment and the amount of 7-HT secreted by the host. Moreover, the specific mechanism by which the SigW/RsiW system regulates the Gac/Rsm system needs to be further explored.

*Pseudomonas* is extremely abundant in various environments. One of the reasons for this strong capability for environmental adaptation is closely related to the fact that they encode a wide variety of ECF σ factors [36,52]. While the SigW/RsiW system is conserved in the group of *P. fluorescens* DNA homologues, it has only been reported to contribute to bacterial oxidative stress and increased drug resistance [44,53,54]; therefore, this work is the first report of the SigW/RsiW system being involved in bacterial iron regulation. 7-HT, a secondary metabolite with toxicity, is important for the pathogenicity and environmental adaptation of *P. donghuensis* HYS. In this study, we systematically elucidated the biological function of this ECF-σ/anti-σ system regarding the regulation of siderophore synthesis, which is a new discovery, not only with respect to the regulatory mechanism of 7-HT biosynthesis but also for the function of σ^70^ family members.

## 4. Materials and Methods

### 4.1. Bacterial Strains and Culture Conditions

Table S1 lists the bacterial strains and plasmids used in this study. *P. donghuensis* HYS obtained from Donghu Lake was used as a parental strain and designated the wild-type. pBBR1MCS-2 and pET41a(+) were used for gene overexpression assays. All strains were stored at −80 °C in LB broth. Plasmids and filter-sterilized antibiotic stock solutions were stored at −20 °C. *E. coli* strains were routinely grown in LB medium at 37 °C. *Pseudomonas* strains were grown in LB medium and iron-deficient MKB medium (2.5 g/L K_2_HPO_4_, 15 mL/L glycerol, pH 7.2, and subsequently supplemented with 2.5 g/L MgSO_4_ and 5 g/L Casamino Acids) at 30 °C. When required, FeSO_4_·7H_2_O was added at a concentration of 30 μM to the MKB medium. When necessary, antibiotics were added, at the following concentrations: for *E. coli*, 10 μg/mL gentamicin and 50 μg/mL kanamycin; for *P. donghuensis* HYS and its derivative strains, 25 μg/mL chloramphenicol and 50 μg/mL kanamycin.

### 4.2. Construction of Deletion Mutants

The deletion mutant strains were constructed using a homologous recombinant knockout method [55]. To construct a deletion plasmid, a PCR product containing a 500-bp region upstream and downstream of the target gene was digested with a primer-specific restriction enzyme and ligated into pEX18Gm gene-replacement vector. Recombinant plasmids were confirmed by sequencing and introduced into HYS and its derivative strains by *E. coli* S17-1 (λpir) ligation. Individual recombinants are selected for resistance to both antibiotics. These transformants were further cultured overnight in 5 mL of antibiotic-free liquid LB medium, allowing a second allelic exchange to occur. Appropriately diluted cultures were plated on LB agar plates supplemented with 10% sucrose and cultured at 30 °C to further screening for the correct gene deletion mutant. The primers and plasmids used in the construction of the mutants are described in detail in Tables S1 and S3.

### 4.3. Siderophore Determination Assays

The characteristic absorption peaks of 7-HT are at 330 and 392 nm in the medium and that of pyoverdine is 405 nm in liquid MKB. The characteristic peaks of the siderophores were identified according to their UV/visible spectra (UV-2450/2550; Shimadzu; Kyoto, Japanese), measuring the absorption spectra of the filtered supernatants of 24 h MKB cultures (normalized to an OD_600_ = 0.5) every 0.5 nm.

The siderophore yield was determined using the following method. Siderophores secreted by bacteria were detected semi-quantitatively using CAS detection solution. A 24 h MKB culture (optical density at 600 nm (OD_600_) adjusted to 1.0) was compared with double-distilled water (ddH_2_O), and the appropriately diluted supernatant was mixed with an equal volume of CAS assay solution. The absorbances at 630 nm of the sample (As) and blank control (Ar) were determined after 1 h of light-proof reaction. The siderophore unit was calculated according to the formula (Ar − As) × 100/Ar% iron content unit [38]. Estimation of pyoverdine production in liquid MKB medium (emitted at 460 nm after 405 nm excitation) was carried out using a fluorescence spectrometer [56].

### 4.4. Biofilm Formation Analysis

Biofilm formation was analyzed as previously described, with slightly modified measurements [57]. In brief, overnight bacteria 1% were inoculated into LB medium supplemented with appropriate antibiotics in borosilicate tubes. After 48 h of growth at 25 °C, the films were stained with 0.1% crystal violet (CV), and impurities were washed with 1× PBS solution after 20 min of staining. For quantification, the film was dissolved in 1 mL of anhydrous ethanol and the absorbance was measured at 600 nm.

### 4.5. RNA Isolation and RT-PCR

A total of 2 mL was collected of the cultures of *P. donghuensis* HYS and its derivative strains during the exponential phase (at an OD_600_ of 0.6) in 5 mL of liquid MKB medium or medium supplemented with 30 μM FeSO_4_·7H_2_O after incubating at 30 °C. The supernatant was removed by centrifugation (13,000× *g*, 2 min) at 4 °C. Total RNA was extracted using TRIzol reagent (Ambion; Austin, TX, USA) according to the manufacturer’s instructions, and genomic DNA was then removed. RNA was transcribed into cDNA using a PrimeScript RT reagent kit with gDNA Eraser (TaKaRa; Kyoto; Japanese). The RNA was stored at −80 °C, and the cDNA was stored at −20 °C as template for the real-time qPCR. Fluorescence quantitative PCR reactions were run on Bio-Rad Cycler (Bio-Rad Laboratories; Hercules; USA; CFX96 Real-Time System C1000 Touch).

### 4.6. Protein Expression and Purification

To express SigW protein, SigW-F/R primers were used to amplify 169 bp of the *sigW* gene fragment from HYS gDNA. The product was digested and inserted into the pET41a(+) plasmid to produce pET41a(+)-*sigW*, and the plasmid was transferred into *E. coli* BL21(DE3). For protein production, *E. coli* cells were grown in LB medium to an OD_600_ of 0.6–0.8 after being induced with 1 mM of isopropyl β-D-thiogalactoside (IPTG) for 14 h at 25 °C and 100 rpm. The cells were harvested and resuspended in binding buffer (10 mM Tris-HCl pH 7.5, 500 mM NaCl, 10% glycerol), then lysed using a JY92-IIDN homogenizer (Xinzhi, Ningbo, China). The lysate supernatant was filtered and purified using a GST-Sefinose (TM) Resin 4FF column (BBI; Shanghai, China), and the target protein was eluted with 10 mM, 33 mM, and 40 mM GSH eluent, respectively. The resulting protein was verified by SDS-PAGE and stored at −80 °C. Western blotting was performed using mouse anti-GST (Abbkine; Wuhan, China) antibody prepared according to standard methods [58]. Goat anti-mouse IgG-HRPs (Abbkine) was used as a secondary antibody. Finally, ECL was used to develop markers (Figure S4).

### 4.7. Promoter Activity Assay

The plasmid pBBR5Z carrying a promoterless *lacZ* gene and a promoter P*nfs* was used to analyze the effect of the target gene on the tested promoter activity [19]. Plasmids pBBR5Z carrying promoters P1, P9, and P12 were electro-transformed into *P. donghuensis* HYS and Δ*sigW* mutants and incubated in MKB medium until 8 h; the promoter activity was assessed by measuring the β-galactosidase activity of the strain [59].

### 4.8. Electrophoretic Mobility Shift Assay

Electrophoretic mobility shift assay was performed with SigW protein and 0.3 μM DNA promoter fragment in 20 μL of gel shift buffer (10 mM Tris-HCl pH 7.5, 150 mM NaCl, 2 mM DTT, 2 mM EDTA, and 10% glycerol). The DNA fragment is a PCR recovery product having a length of 190 to 250 bp, including the promoter. The primers used in the process are shown in Table S3. The samples were incubated in a PCR apparatus at 30 °C for 40 min and then loaded onto a 6.8% native polyacrylamide gel, which was run on ice for 2.5 h at 100 V in a pre-cooled 0.5 × TBE buffer and visualized with EB Nucleic Acid Stain using a Tanon 5200 Image analysis system (Tanon Technologies, Shanghai, China).

### 4.9. 5′RACE PCR Analysis to Identify Transcription Start Sites

The TSS of *sigW* was identified using a 5′ RACE kit (Vazyme; Nanjing, China) according to the manufacturer’s manual. RNA from wild-type HYS was isolated as described above. Complete removal of DNA contamination was confirmed using RT-PCR. Approximately 100 μg of RNA was reverse-transcribed with gene-specific primers, and nested PCR was performed to obtain the PCR products, which were subsequently cloned into the pMD-19T. The T_m_ temperature for the two primers was 72 °C and 69 °C, respectively. The product of 500 bp in size was selected for cloning and sequencing based on the position of the two GSP primers. Multiple constructs were selected and subjected to DNA sequencing and sequence analysis to identify the TSS.

### 4.10. Bioinformatic Analysis

The σ factor analysis of prokaryotic genes was performed using the P2TF database (http://www.p2tf.org/ accessed on 30 April 2022). The nucleic acid sequences of 35 σ factors—most of which have been experimentally verified—were downloaded from the NCBI public database.

PromPredict (http://nucleix.mbu.iisc.ernet.in/prompredict/prompredict.html accessed on 20 January 2022), a web-based tool, was used to identify promoter regions in genomic DNA.

InterProScan (https://www.ebi.ac.uk/interpro/ accessed on 1 March 2022) website was used to analyze conserved structural domains in protein sequences.

RNA-Seq was conducted by the Shanghai Majorbio Bio-pharm Technology Company.

The MEME Suit website (https://meme-suite.org/meme/tools/meme accessed on 26 October 2022) is used to predict conserved motifs of protein sequences by uploading 20 amino acid sequences with >80% identity to the SigW, outputting the results and then setting the motif with a significance level of E value < 0.05 for analysis.

Prediction of the promoter region of the manipulator and the corresponding −10 and −35 regions was made using the online tool BPROM on the Softberry website (http://www.softberry.com/ accessed on 8 October 2022).

### 4.11. Accession Numbers

*P. donghuensis* HYS whole-genome shotgun contigs were deposited in the NCBI database (accession no. NZ_AJJP00000000). The GenBank accession numbers for the genes *sigW* and *rsiW* from *P. donghuensis* HYS are UW3_RS0125380 and UW3_RS0125385, respectively, accessed on 4 May 2022.

## Data Availability

All data generated or analyzed during this study are included in this published article (and its Supplementary Information files).

## Supplementary Materials

The following supporting information can be downloaded at: https://www.mdpi.com/article/10.3390/ijms24021184/s1. References [19,60] are cited in the Supplementary Materials.
