# Supplementary material for: Insight into the Global Negative Regulation of Iron Scavenger 7-HT Biosynthesis by the SigW/RsiW System in Pseudomonas donghuensis HYS
Source: Int J Mol Sci. 2023 Jan 7;24(2):1184. doi: 10.3390/ijms24021184 (PMC9861184; doi:10.3390/ijms24021184)
Supplement: Supplementary file 1 [file ijms-24-01184-s001.zip › ijms-2047436-supplementary.pdf]

International Journal of *Molecular Sciences*

MDPI

Supplementary Materials

Table S1. Bacterial strains and plasmids used in this study

Strains and Plasmids	Description	Source
Escherichia coli		
DH5α	F-*φ80lacZ ΔM15 Δ(lacZYA-argF) U169 recA1 endA1 hsdR17(rk-, mk+) phoA supE44 thi-1 gyrA96 relA1 tonA*	Laboratory stock
BL21	F-*ompT hsdSB* (*rB-mB-*) *gal dcm* (DE3)	Laboratory stock
S17-1 λpir	*thi pro hsdR recA*; chromosomal RP4–2; (Tc::Mu) (Km::Tn7) Tp^r Sp^r	Laboratory stock
Pseudomonas donghuensis		
HYS	*P.donghuensis* wild-type strain	Laboratory stock
Δ*sigW*	*sigW* derivative of HYS	This study
Δ*rsiW*	*rsiW* derivative of HYS	This study
Δ*sigW*Δ*rsiW*	*sigW* and *rsiW* derivative of HYS	This study
Δ*sigW*Δ*rsiW* (pBBR1-MCS2-*sigW*)	*sigW* overexpression of *sigW and rsiW* deletion mutant	This study
HYS/pBBR1-MCS2	pBBR2 expression of HYS	This study
HYS/pBBR1-MCS2-*sigW*	*sigW* overexpression of HYS	This study
HYS/pBBR1-MCS2-*rsiW*	*rsiW* overexpression of HYS	This study
HYS/pBBR5Z-P*orf9-6*	HYS containing pBBR5Z-P*orf9-6*	Yu, 2014[1]
HYS/pBBR5Z-P*orf1*	HYS containing pBBR5Z-P*orf1*	Yu, 2014[1]
HYS/pBBR5Z-P*orf12*	HYS containing pBBR5Z-P*orf12*	Yu, 2014[1]
Δ*sigW*/pBBR5Z-P*orf9-6*	Δ*sigW* containing pBBR5Z-P*orf9-6*	This study
Δ*sigW*/pBBR5Z-P*orf1*	Δ*sigW* containing pBBR5Z-P*orf1*	This study
Δ*sigW*/pBBR5Z-P*orf12*	Δ*sigW* containing pBBR5Z-P*orf12*	This study
Plasmids		
pBBR1-MCS2	Ka^R, oriREP, gene expression vector	Laboratory stock
pET41a(+)	Ka^R, ori ColE1/pMB1/pBR322/pUC, gene expression vector	Laboratory stock
pBBR1-MCS2-*sigW*	*sigW* overexpression in the pBBR1-MCS2, Ka^R	This study
pBBR5Z-P*orf9-6*	*Porf9-6'-'lacZ* transcriptional fusion cloned in pBBR5Z; Gm^R	Yu, 2014[1]
pBBR5Z-P*orf1*	*Porf1'-'lacZ* transcriptional fusion cloned in pBBR5Z; Gm^R	Yu, 2014[1]
pBBR5Z-P*orf12*	*Porf12'-'lacZ* transcriptional fusion cloned in pBBR5Z; Gm^R	Yu, 2014[1]
pET41a(+)-SigW	Ka^R, Insert a *sigW* fragment without stop codon between BamHI and HindIII sites of pET41a(+)	This study
pEX18Gm-*sigW*	Gm^R, Gene replacement vector for *sigW*	This study

Table S2. The whole genome sequence of *P. donghuensis* HYS was uploaded to the P2TF database. Genome-wide search for transcription factors was performed, and σ factor was selected as the screening category with a threshold of 80%[2]. Information of the resulting 35 σ factors is shown in the following table, with 20 of them named *σ1* to *σ19* and *sigW*, respectively.

Table S2. The σ factors of *P. donghuensis* HYS

Gene ID	Gene Name	Identity(%) (P2TF)	Type	Description
UW3_RS0101765		81.6	Unclassified	sigma-70 family RNA polymerase sigma factor
UW3_RS0108825	*σ1*	82.3	ECF	sigma-70 family RNA polymerase sigma factor
UW3_RS0125380	*sigW*	84.3	ECF	sigma-70 family RNA polymerase sigma factor
UW3_RS0113790		83.3	Unclassified	RNA polymerase sigma factor
UW3_RS0114990	*σ2*	88.9	ECF	RNA polymerase sigma factor SigX
UW3_RS0120050	*fliA*	88.5	RpoD	RNA polymerase sigma factor FliA
UW3_RS0115575	*σ3*	86.5	ECF	sigma-70 family RNA polymerase sigma factor
UW3_RS0124180	*σ4*	84.7	ECF	RNA polymerase sigma factor
UW3_RS0119060	*σ5*	80.1	ECF	sigma-70 family RNA polymerase sigma factor
UW3_RS0106190	*rpoD*	92.3	RpoD	RNA polymerase sigma factor RpoD
UW3_RS0115845	*σ6*	80	ECF	RNA polymerase sigma factor
UW3_RS0125395		84.2	Unclassified	sigma-70 family RNA polymerase sigma factor
UW3_RS0109580	*σ7*	82.1	ECF	sigma-70 family RNA polymerase sigma factor
UW3_RS0119320	*rpoS*	87	RpoD	RNA polymerase sigma factor RpoS
UW3_RS0116405		88.9	Unclassified	sigma-70 family RNA polymerase sigma factor
UW3_RS0100545	*σ8*	86	ECF	sigma-70 family RNA polymerase sigma factor
UW3_RS0113120	*σ9*	81.8	ECF	sigma-70 family RNA polymerase sigma factor

UW3_RS0113745	*σ10*	82.1	ECF	RNA polymerase sigma factor
UW3_RS0118685		100	Unclassified	sigma-70 family RNA polymerase sigma factor
UW3_RS0103900	*σ11*	90.5	ECF	RNA polymerase sigma factor RpoE
UW3_RS0115310		89.8	RpoN	RNA polymerase factor sigma-54
UW3_RS0121460		82.7	Unclassified	sigma-70 family RNA polymerase sigma factor
UW3_RS0103755		83.9	Unclassified	sigma-70 family RNA polymerase sigma factor
UW3_RS0124665	*σ12*	83.8	ECF	FecR domain-containing protein
UW3_RS0106005	*σ13*	84.8	ECF	RNA polymerase sigma factor
UW3_RS0125045		90.9	Unclassified	Terminase family protein
UW3_RS0102655	*σ14*	90.8	ECF	sigma-70 family RNA polymerase sigma factor
UW3_RS0125850	*topA*	95.3	Unclassified	type I DNA topoisomerase
UW3_RS0121090	*σ15*	83.7	ECF	sigma-70 family RNA polymerase sigma factor
UW3_RS0103735	*σ16*	89	ECF	sigma-70 family RNA polymerase sigma factor
UW3_RS0124670	*σ17*	83.8	ECF	RNA polymerase sigma factor
UW3_RS0122855	*σ18*	88.6	ECF	RNA polymerase factor sigma-70
UW3_RS0102630		85.3	Unclassified	sigma-70 family RNA polymerase sigma factor
UW3_RS0116805		82.5	Unclassified	DUF4880 domain-containing protein
UW3_RS0109705	*σ19*	91	ECF	RNA polymerase sigma factor RpoH

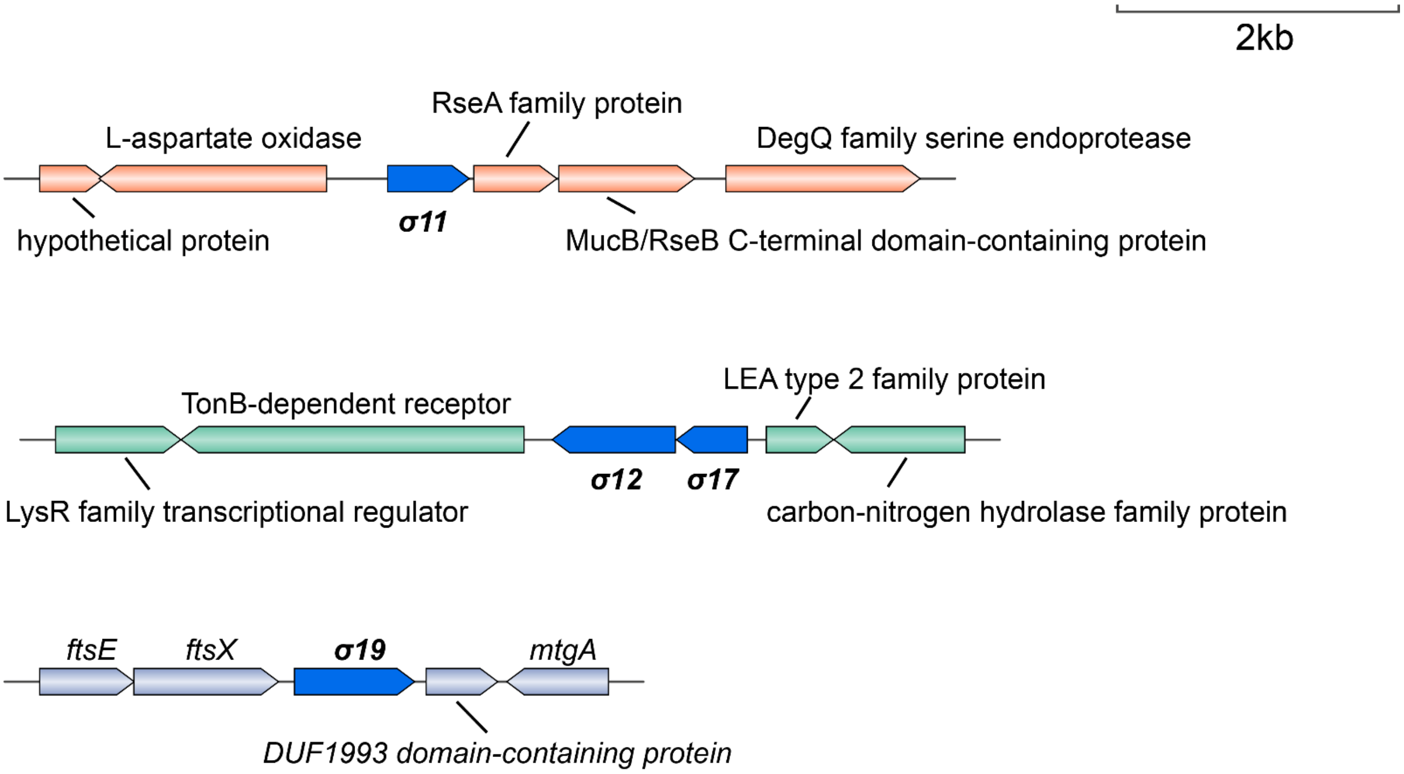

Figure S1. Genomic localization map of ECF σ factors regulated by ferrous ions. From top to bottom, the distribution of *σ11*, *σ12*, *σ17*, and *σ19* on the genome of *P. donghuensis* HYS.

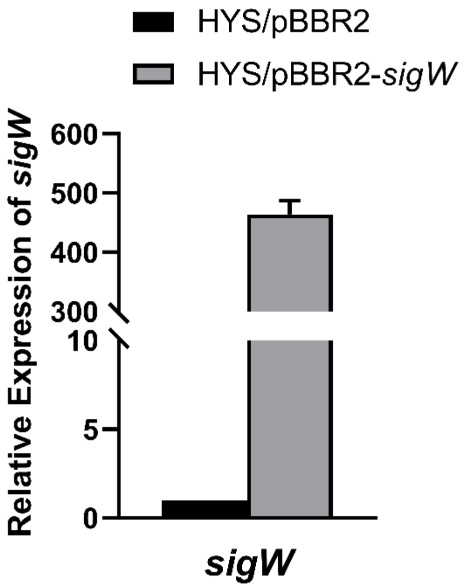

Figure S2. Relative expression of *sigW* in HYS/pBBR1-MCS2 and HYS/pBBR1-MCS2-*sigW*. RNA was isolated from the indicated strains grown to the exponential phase at 30 °C in liquid MKB culture. The error bars indicate the mean ± SD of three independent experiments.

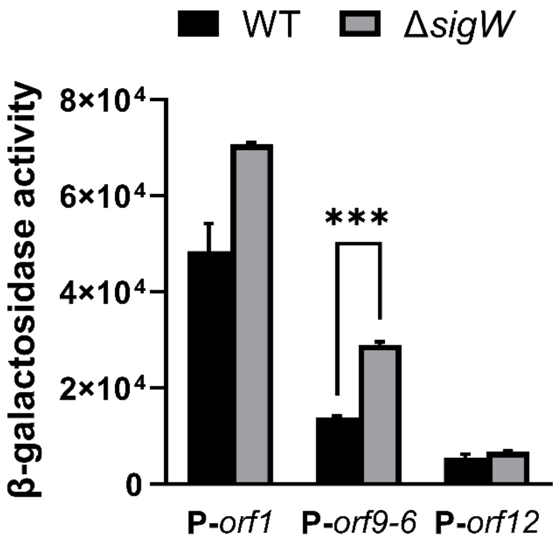

Figure S3. SigW is the transcriptional inhibitor of the nonfluorescent siderophore *(nfs)* gene cluster. The promoter activity of *orf1*, *orf9–6*, and *orf12* were measured in wild-type HYS and Δ*sigW* mutant. β-galactosidase activities of the recombinant HYS strains were detected when the *lacZ* gene was driven by various promoters in the pBBR5Z vector. Error bars indicate the mean ± SD of three independent experiments. Statistical significance was calculated using one-way ANOVA Dunnett's multiple comparison test, ***$p < 0.0001$.

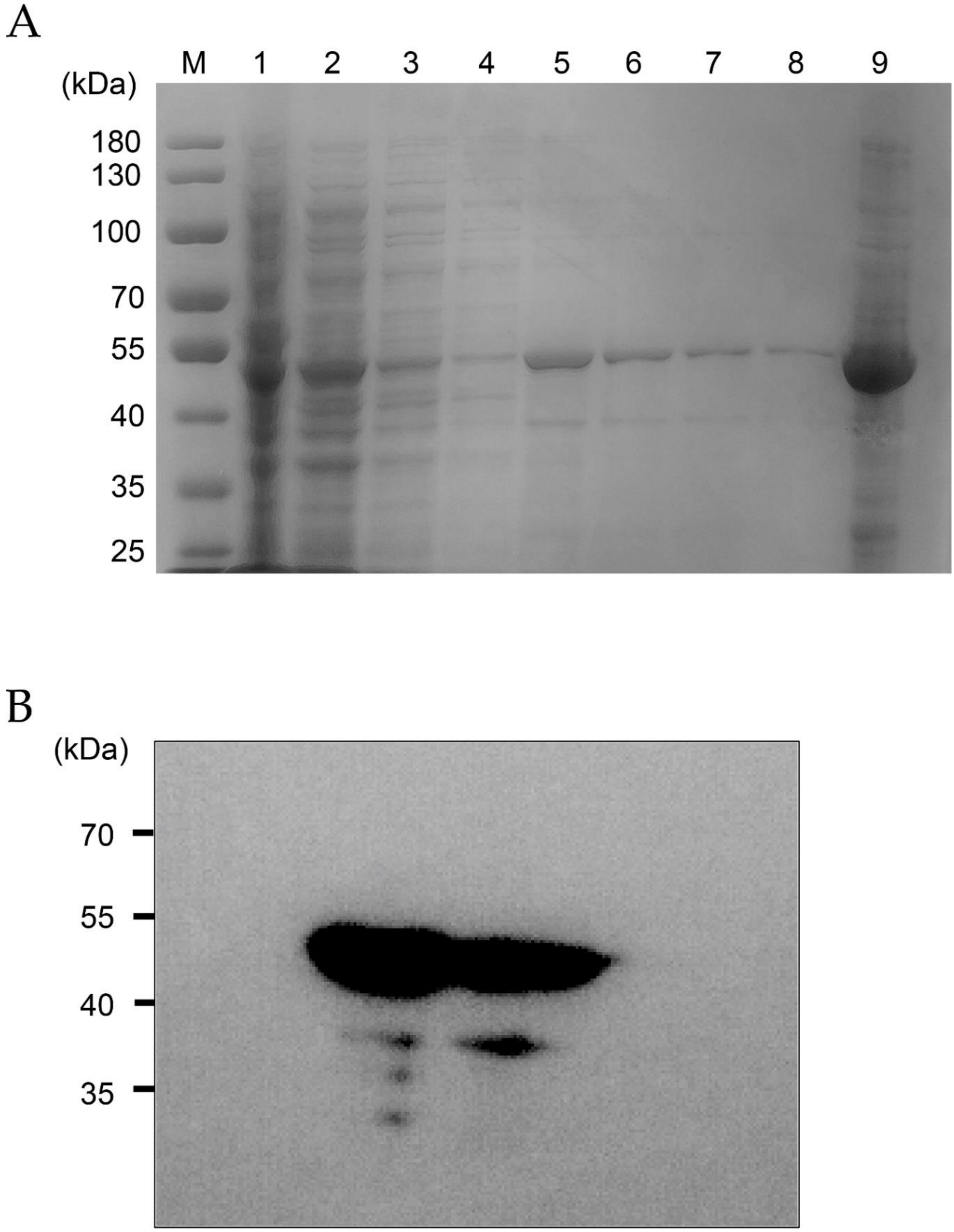

Figure S4. Recombinant protein purification. (A) SDS–PAGE analysis of the purified SigW. Marker: Pre-stained Protein Ladder 26616 (Thermo Fisher, USA). Lanes 1–9 are cellular sedimentation, supernatant, wash buffer I, wash buffer II,10 mM GSH, 33 mM GSH, 40 mM GSH, 40 mM GSH, and Ultrafiltrate protein, respectively; and (B) Western blot analysis of SigW expressed in *E.coli* BL21(DE3). The fusion protein GST-SigW in strain BL21 carrying the plasmid pET41a(+)-SigW was detected by Western blot method with an anti-GST antibody(Abbkine) at 14 h after IPTG induction.

Table S3. Oligonucleotide primers used in this study

Primer	Primer Sequence (5′-3′)	Description
RT-*sigW*-F	GGGCAGCAACACCGAAGATA	Real-time qPCR
RT-*sigW*-R	GCGACAAGCGTGACATTACC	Real-time qPCR
RT-*rsiW*-F	TCGGCACTTTACCGGACCTCTC	Real-time qPCR
RT-*rsiW*-R	GGGCGTCTTCAAACAACAACAGC	Real-time qPCR
RT-*σ1*-F	CACGCCTTTATCCTGCATCG	Real-time qPCR
RT-*σ1*-R	CTCAGGCAATGCACGAAGG	Real-time qPCR
RT-*σ2*-F	GCCTTGAGTCTTGATCCGCT	Real-time qPCR
RT-*σ2*-R	TCGTAGCACCAGAATCTCGC	Real-time qPCR
RT-*σ3*-F	TCGTTTTCCAGTACCCGCAC	Real-time qPCR
RT-*σ3*-R	GGCTTTCTCGACCACTACCA	Real-time qPCR
RT-*σ4*-F	TAGAGATAGGCCGGGGTGTT	Real-time qPCR
RT-*σ4*-R	TTCCTCAAGCACGCGAAAAC	Real-time qPCR
RT-*σ5*-F	TGCTCTATGTCATTCCCGCC	Real-time qPCR
RT-*σ5*-R	TCGCGAACTGGTGAGTTTCC	Real-time qPCR
RT-*σ6*-F	GCAAGTGCTCGCAACCTTAC	Real-time qPCR
RT-*σ6*-R	CACCGTGCTCAGAGAAACCT	Real-time qPCR
RT-*σ7*-F	CGGCCTTCCTCTTCACCATT	Real-time qPCR
RT-*σ7*-R	CCTGGATAGCCTCGTGCATC	Real-time qPCR
RT-*σ8*-F	CGGATCTTCATCCTCAGCCG	Real-time qPCR
RT-*σ8*-R	AGTTCCTTCTGAACCGTGCT	Real-time qPCR
RT-*σ9*-F	CGAATAGTGTCGGCATTGCG	Real-time qPCR
RT-*σ9*-R	GTTGAAGAACTGGCTGCGTG	Real-time qPCR
RT-*σ10*-F	TCTGCTTGTAGCGGGTCTTG	Real-time qPCR

RT-*σ10*-R	CGATGAGGCGGTACAGGATG	Real-time qPCR
RT-*σ11*-F	TGTAAGTTCCGAGGATGCGG	Real-time qPCR
RT-*σ11*-R	TGCCTTCGATCTCATCTCGC	Real-time qPCR
RT-*σ12*-F	GTAGCGTTGTTGGTCGCTG	Real-time qPCR
RT-*σ12*-R	GCGATTTGTCCGATGATGCC	Real-time qPCR
RT-*σ13*-F	ATCAACTCGGCCAGATACGC	Real-time qPCR
RT-*σ13*-R	CTGAGCCAGGACACTTTCGT	Real-time qPCR
RT-*σ14*-F	CCAGGAGTTGTTTTTGCGCT	Real-time qPCR
RT-*σ14*-R	GATCAATGGCGATGTTGCCG	Real-time qPCR
RT-*σ15*-F	ATCTTCGACAGCCTGATGGC	Real-time qPCR
RT-*σ15*-R	TTCGGCGGTTTTCTCGTAGG	Real-time qPCR
RT-*σ16*-F	CACGTCCTGGGTCATGTCTG	Real-time qPCR
RT-*σ16*-R	AACCTGTCGCGGGTTCTATG	Real-time qPCR
RT-*σ17*-F	GCTGCTCGATCTTCACCAGT	Real-time qPCR
RT-*σ17*-R	ATGTTGACGTCTCCCGTGTC	Real-time qPCR
RT-*σ18*-F	ACATCAACTTCGCCACCCTT	Real-time qPCR
RT-*σ18*-R	GATGTCTTTCTGTGGCACGC	Real-time qPCR
RT-*σ19*-F	GAAGTGCGGGAGATGGAGAG	Real-time qPCR
RT-*σ19*-R	TTGTCACTCCAGTCGGCATC	Real-time qPCR
RT-*rpoB*-F	CGGGAGCGACCAAAGATCAG	Real-time qPCR
RT-*rpoB*-R	CGTACTCCAGGGCAGCATTG	Real-time qPCR
RT-*orf1*-F	TTCGTTCCGCATCCCCAT	Real-time qPCR
RT-*orf1*-R	AACACTCCTTGAGCGTCTGG	Real-time qPCR
RT-*orf2*-F	CCGCTGCCGTTCTTCAAATTGC	Real-time qPCR

RT-*orf2*-R	GATCACCGCCGAAAGCACTACC	Real-time qPCR
RT-*orf3*-F	CTTGCCCTCGATCTTCGGTT	Real-time qPCR
RT-*orf3*-R	GTTATCCTCGACCAGCACCC	Real-time qPCR
RT-*orf4*-F	CAACCAGGAGCAGTGGGTAG	Real-time qPCR
RT-*orf4*-R	AAGGCGCAAAAGATTGCCAG	Real-time qPCR
RT-*orf5*-F	GGGGATCTTCGAACCCAAGG	Real-time qPCR
RT-*orf5*-R	TGGTGCCGTAGTGAACCATC	Real-time qPCR
RT-*orf6*-F	CATGCGTTCCATCACCACTTCAATG	Real-time qPCR
RT-*orf6*-R	CAACTGCCACAGGTGCTCGATAC	Real-time qPCR
RT-*orf7*-F	GATGATGTCTGGCTGGAAGTCCTTG	Real-time qPCR
RT-*orf7*-R	GAAACTCGGCTGCACGGTGATC	Real-time qPCR
RT-*orf8*-F	GTAAAGCCCCACCAGTCCTC	Real-time qPCR
RT-*orf8*-R	GAAACTCTCCTGGACTCCGC	Real-time qPCR
RT-*orf9*-F	GAATGCTTGCGGTACAGGGATGG	Real-time qPCR
RT-*orf9*-R	AAGTCGTGAAGTTCGGCACCAAG	Real-time qPCR
RT-*orf10*-F	AGCTTGAACAACTGCATGGC	Real-time qPCR
RT-*orf10*-R	TGGTGCCACTGACGAAAGAA	Real-time qPCR
RT-*orf11*-F	CCTGTGGCTACGAAGACCTTGAAG	Real-time qPCR
RT-*orf11*-R	CCAGGCGAGTGATCTGCTTGTC	Real-time qPCR
RT-*orf12*-F	TACACCGAGCCGACACTAACCC	Real-time qPCR
RT-*orf12*-R	TCGAGACCATCCTCAAGACTACCG	Real-time qPCR
RT-*gacA*-F	CTTGCGGCTGGAACGACTTGAG	Real-time qPCR
RT-*gacA*-R	GGTGCAGGGCTGGATGAAATGG	Real-time qPCR
RT-*gacS*-F	GACAGCTCGATTTCCACCCA	Real-time qPCR

RT-*gacS*-R	ATGACGCCACCCGTTATCTG	Real-time qPCR
RT-*rsmY*-F	TGCAGACTGTTTCCCTGACATC	Real-time qPCR
RT-*rsmY*-R	CTTCTTACATGGACGTAGCGCA	Real-time qPCR
RT-*rsmZ*-F	ACTGACACAGGCTTTCAAGGATGAG	Real-time qPCR
RT-*rsmZ*-R	TTCCGTAGTCCCTTTGTTCCCTTTC	Real-time qPCR
p1-F	TCAATGGCGATCAGTACCGAGC	EMSA
p1-R	TATCCGGAAAGTGCATGGTAAGCA	EMSA
p2-F	GATGCTTTTGAGCGCTTATATATACC	EMSA
p2-R	CGAACTCATGGCCTGCCC	EMSA
p9-F	GCCCGCGTATTTCCTGAGC	EMSA
p9-R	CGGTGTCAAAACCCTTGTTGC	EMSA
p10-F	GGGCTTGCTGGGTTTTAAATGG	EMSA
p10-R	ATCAACTGCACTCCTTGAGGC	EMSA
p12-F	CCCTACCGTATTTGAACGGC	EMSA
p12-R	CCTCGAGCGGGCTTGTGT	EMSA
GSP1	GCGCGGGCGCGCGACAAGCGTGACATTA	5′RACE
GSP2	GCGCGCAGGCTGGCGTCTTCGCGTTGTT	5′RACE
SigW-F	CG<u>GGATCC</u>AATGAACGAACTAGAC	SigW cloning
SigW-R	CCC<u>AAGCTT</u>TTTCAACCTCCG	SigW cloning
sigW-up-F	CGGGATCCACTTGCTGGTAGAA	Constructing Δ*sigW*
sigW-up-R	CGGAATTCTAGTTCGTTCATTGC	Constructing Δ*sigW*
sigW-down-F	CGGAATTCAAATGAGCTTGA	Constructing Δ*sigW*
sigW-down-R	CCAAGCTTGTAGGCCGAGA	Constructing Δ*sigW*
rsiW-up-F	CGGAATTCGCCGGTACGA	Constructing Δ*rsiW*

rsiW-up-R	CGGGATCCGATCAAGCTCATTTC	Constructing Δ*rsiW*
rsiW-down-F	CGGGATCCATCACTTTGTAGCTG	Constructing Δ*rsiW*
rsiW-down-R	CCCAAGCTTGAGATTGATTCT	Constructing Δ*rsiW*
sigW-M-F	GCTGGCGTAGGTCGTGTTG	Verification of *sigW* knockout
sigW-M-R	CTTACGCCCCTAATGGCA	Verification of *sigW* knockout
rsiW-M-F	GCCCTTGGCGTGATTCTTA	Verification of *rsiW* knockout
rsiW-M-R	CACCTGGATGGATTAGACTCAA	Verification of *rsiW* knockout

[a] Restriction sites displayed in underline.

1. Yu, X.; Chen, M.; Jiang, Z.; Hu, Y.; Xie, Z. The two-component regulators GacS and GacA positively regulate a nonfluorescent siderophore through the Gac/Rsm signaling cascade in high-siderophore-yielding *Pseudomonas* sp. strain HYS. *J Bacteriol* **2014**, *196*, 3259-3270. https://dx.doi.org/10.1128/JB.01756-14
2. Ortet, P.; De Luca, G.; Whitworth, D.E.; Barakat, M. P2TF: a comprehensive resource for analysis of prokaryotic transcription factors. *BMC genomics* **2012**, *13*, 628-636. https://dx.doi.org/10.1186/1471-2164-13-628
